# The effectiveness of nursing interventions on fatigue and sleep quality in hospitalized cancer patients: the role of foot massage and bed bath

**DOI:** 10.1007/s00520-026-10386-7

**Published:** 2026-02-05

**Authors:** Ayşe Kabuk, Ufuk Demirel, Demet Inangil

**Affiliations:** 1 Department of Nursing, Faculty of Health Sciences, Zonguldak Bülent Ecevit University, Zonguldak, Türkiye; 2 Fundamental of Nursing Department, Hamidiye Faculty of Nursing, University of Health Sciences, Istanbul, Turkey

**Keywords:** Fatigue, Foot massage, Bed bath, Cancer nursing, Sleep quality

## Abstract

**Background:**

In diseases requiring long-term treatment, such as cancer, the importance of holistic nursing support is increasing. Both foot massage and bed baths are holistic care methods that address patients' physical, emotional, and psychological needs, and research indicates they can effectively reduce symptoms like fatigue and sleep disturbances.

**Objective:**

This study aims to evaluate and compare the effects of foot massage and bed baths on fatigue and sleep quality in hospitalized cancer patients.

**Method:**

The research was conducted with hospitalized patients in the oncology clinic of a university hospital between April and November 2024, employing a three-group randomized controlled experimental design. The study included 39 cancer patients who were randomly assigned to three groups: Foot Massage (FM) (*n* = 12), Bed Bath (BB) (*n* = 12), and Control (*n* = 15). The treatments were administered for four consecutive days, two hours before bedtime, with each session lasting 30 min. The control group received routine care without additional interventions. Data was collected using the Patient Information Form, the Brief Fatigue Inventory (BFI), and the Richard Campbell Sleep Questionnaire (RCSQ), through five repeated measurements.

**Results:**

Before the interventions began, there were no significant differences between the groups in terms of fatigue or sleep quality. Regarding the BFI scores, on days three (*p* < .05) and four (p < .001) the FM group demonstrated lower mean scores compared with both the BB group and the control group. On day five, the BFI scores of the FM group and the BB group remained lower than those of the control group, and this difference reached statistical significance (*p* < .001). Regarding the RCSQ scores, starting from day three, the FM group exhibited significantly higher mean scores than the control group (*p* < .05 and *p* < .001). From day four onward, the BB group also demonstrated significantly higher RCSQ scores compared with the control group (*p* < .001). No significant differences were observed between the FM and BB groups regarding the RCSQ (p > .05).

**Conclusion:**

These findings suggest that foot massage and bed baths serve as effective supplementary nursing interventions for reducing fatigue and improving sleep quality in hospitalized cancer patients.

**Clinical trial number:**

NCT06373614.

**Trial registration:**

ClinicalTrials. gov Registry (NCT 06373614) in April 2024.

**Supplementary Information:**

The online version contains supplementary material available at 10.1007/s00520-026-10386-7.

## Highlights

- Evidence remains limited regarding the effectiveness of foot massage and bed baths in reducing fatigue and enhancing sleep among cancer patients.
- Foot massage significantly decreased fatigue starting from day three compared to both the bed bath and control groups.
- Both interventions lowered fatigue levels and improved sleep quality by day five.
- These non-pharmacological nursing approaches are feasible, acceptable, and effective in hospital settings for cancer care.

**Supplementary Information:**

The online version contains supplementary material available at 10.1007/s00520-026-10386-7.

## Introduction

Cancer is one of the leading health issues with the highest mortality and morbidity rates worldwide, contributing to one in every six deaths, resulting in 9.6 million deaths in 2018 [1]. According to GLOBOCAN 2022 estimates published by the International Agency for Research on Cancer [2], approximately 20 million new cancer cases and 9.7 million cancer-related deaths occurred worldwide in 2022, and the global cancer burden is projected to continue rising in the coming decades [3]. Cancer and its treatment methods cause physical and psychological side effects, including pain, loss of appetite, cachexia, nausea, fatigue, sleep disorders, depression, and anxiety in most patients [4, 5].

Cancer-related fatigue is the most common symptom after pain [6], with a reported prevalence of 52% [7]. Fatigue is a subjective experience, and patients are 20 times more likely to report severe fatigue than what physicians would expect [8]. There are numerous causes of cancer-related fatigue, including chemotherapy, insomnia, being female, and depression, all of which increase the risk [7]. Additionally, sleep issues are common among cancer patients, with 50% experiencing disorders such as insomnia [9]. Cancer-related fatigue correlates with sleep quality; individuals with cancer who have poor sleep quality tend to feel more fatigued [10]. Consequently, fatigue and insomnia negatively impact patients' quality of life concerning functional independence, muscle strength, mood, and social relationships, making it challenging for individuals to maintain their daily routines [11, 12]. This situation diminishes patients' quality of life and can also lead to cognitive decline [9, 13].

During cancer treatment, managing these symptoms is crucial in nursing care practices. Foot massage emerges as a notable relaxing technique among nursing interventions, helping to alleviate muscle spasms by improving blood circulation [14, 15]. The literature indicates that both classical foot massage and reflexology can significantly reduce fatigue, pain, and anxiety levels in cancer patients [15, 16]. Likewise, bed baths are fundamental to nursing care, offering hygiene along with relaxation, safety, and psychological support. Studies have found bed baths effective in reducing fatigue, and patients express satisfaction with the techniques used during bathing [17–20]. Due to the infection risk linked with traditional tub baths, it has been noted that baths conducted with disposable wet wipes are safer [17–22].

In diseases demanding long-term treatment, such as cancer, the importance of holistic nursing support is on the rise. This is particularly true for inpatients, as short-term supportive care practices are vital; comfort during hospitalization directly influences well-being. Both foot massage and bed baths represent holistic care techniques that engage the physical, emotional, and psychological aspects of individuals, and research shows they effectively reduce symptoms like stress, sleep disorders, and fatigue [15–23]. This study aims to evaluate and compare the effects of foot massage and bed baths on fatigue and sleep quality in hospitalized cancer patients. The lack of studies directly comparing these two practices in the literature renders this research unique and contributes to nursing knowledge by supporting evidence-based care. It should be noted that this study focuses on the short-term effects of these interventions.

## Research Hypotheses (H1)

### Foot massage group

- H1^1^: Foot massage reduces fatigue levels in hospitalized cancer patients.
- H1^2^: Foot massage improves sleep quality in hospitalized cancer patients.

### Bed bathing group

- H1^3^: Bed bathing reduces fatigue levels in hospitalized cancer patients.
- H1^4^: Bed bathing improves sleep quality in hospitalized cancer patients.

### Comparative effects

- H1^5^: There is a significant difference in fatigue levels between cancer patients who receive foot massage and those who receive bed bathing.
- H1^6^: There is a significant difference in sleep quality between cancer patients who receive foot massage and those who receive bed bathing.

## Material and methods

### Study design

This clinical trial was a three-group randomized controlled experimental study and was conducted in accordance with the Consolidated Standards of Reporting Trials (CONSORT) checklist. The study protocol has been registered on clinicaltrials.gov (NCT06373614, Registration Date: 2024–04–15).

### Variables of the study

The independent variables are foot massage and bed bathing practices. The dependent variables are sleep quality and fatigue level.

### Participants

**Fig. 1:**
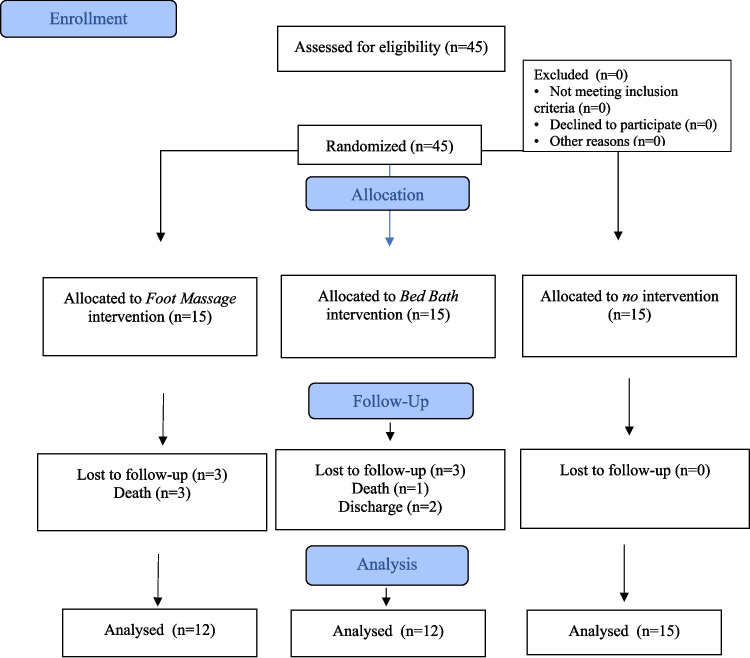
Flow chart of the study (CONSORT 2010)

The research was conducted with hospitalized patients in the oncology clinic of a university hospital between April and November 2024. Inclusion criteria for the study were individuals aged 18 and older, who are literate, willing to participate in the study, have been diagnosed with cancer, patients with no open wounds on their feet, those with no limb loss, individuals not using sleep medication, and patients receiving treatment in a hospital setting. Individuals who were unable to receive the research intervention within five days or failed to participate within the specified time frame were excluded from the study; this occurred because four patients died and two were discharged during the intervention period. The study was completed with a total of 39 participants: 12 in the FM group, 12 in the BB group, and 15 in the control group (Fig. 1.). The sample size was calculated using G*Power 3.1.9.7 software. For the calculation, a mixed-design ANOVA was conducted with 5 measurement points, 3 groups, an effect size of 0.25 (d = 0.25), a 5% margin of error (Œ ± = 0.05), and a power of 95% (1-Œ ≤ = 0.95), resulting in a total sample size of 39 [24, 25].

### Randomization

Participants were assigned randomly to three groups using random.org: Foot Massage (FM), Bed Bathing (BB), and Control (C).

### Ethics approval

The protocol for this study has been approved by the Non-Interventional Clinical Research Ethics Committee of Zonguldak Bülent Ecevit University, with approval number 2023/18, dated 04/10/2023. Written institutional permission for data collection was obtained from the administration of Zonguldak Bülent Ecevit University Hospital (number: E-16734702–622.03–373592 date: 06/11/2023). Informed consent was obtained from all individual participants included in the study. This research was conducted in accordance with the Declaration of Helsinki.

### Data collection forms

Data for the study were collected using the Patient Information Form, the Brief Fatigue Inventory, and the Richard Campbell Sleep Scale.

#### Patient information form

The researchers prepared this form based on the literature [1–3, 26]. The form was shared with three nurses who have Ph.D. degrees. The form was finalized after taking the nurses' opinions into account. It includes a total of 14 questions, consisting of 10 related to the participants' sociodemographic characteristics and 4 concerning their medical status. The form was administered to all patients once at the beginning of the study.

#### Brief fatigue inventory (BFI)

The Brief Fatigue Inventory, developed by the MD Anderson Cancer Center, has been used to assess fatigue levels in cancer patients [27]. Mendoza and colleagues reported a Cronbach's α value of 0.96 for the scale. In a study conducted in Turkey by Azak and Çınar (2005), this value was reported as 0.98 [28]. The BFI assesses the general level of fatigue and the impact of daily living activities over the past 24 h. The overall fatigue score is computed by dividing the total score of items 1, 2, and 3 by 3, while the impact score on daily living activities is calculated by summing the scores of six items (4a, 4b, 4c, 4 d, 4e, 4f) and dividing by 6. The scores obtained from the BFI are interpreted as follows: 0 = no fatigue, 1–3 = low level, 4–6 = moderate level, 7–9 = high level, and 10 = maximum level of fatigue [29]. In this study, the BFI was administered to all patients daily for five consecutive days, and item mean scores were used for the analysis. The Cronbach's α values for the scale over the 5 days were 0.987 on the first day, 0.99 on the second day, 0.989 on the third day, 0.990 on the fourth day, and 0.988 on the fifth day.

#### Richard campbell sleep questionnaire (RCSQ)

Developed by Richard in 1987, this scale has a Cronbach's α value of 0.82. The Turkish validity and reliability study of the scale was conducted by Özlü and Özer in 2015 [30, 31]. The scale consists of 6 items that evaluate the depth of nighttime sleep, time taken to fall asleep, frequency of awakenings, duration of wakefulness after awakening, sleep quality, and ambient noise levels. Each item is assessed using a visual analog scale ranging from 0 to 100. The total score of the scale is calculated by summing the scores of the 5 items; the 6th item, which assesses the noise level in the environment, is not included in this calculation. As the scale score increases, it is believed that the patient's sleep quality improves as well [30, 31]. In this study, the RCSQ was administered daily to all patients for five consecutive days, and the analysis was based on item mean scores, with higher averages indicating better sleep quality. The Cronbach's α values of the scale over the 5 days were found to be 0.951 on the first day, 0.850 on the second day, 0.962 on the third day, 0.988 on the fourth day, and 0.977 on the fifth day.

### Data collection procedure

**Fig. 2:**
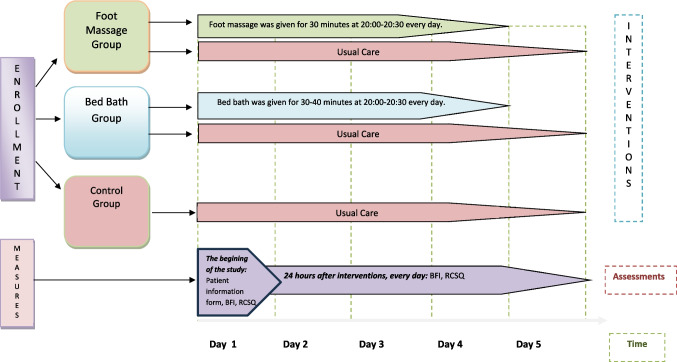
Procedures flow diagram

In the clinic where the study was conducted, all rooms are single-occupancy, and each patient is accompanied by a caregiver. There is no routine practice of providing foot massages or bed baths in the clinic's standard care protocol. Patients' self-care needs are addressed either by themselves or by their caregivers. At the study's beginning, each participant was informed about the study and provided with a written example of this information. Once the individual verbally expressed their willingness to participate, written and signed consent was obtained, and the Patient Information Form, Brief Fatigue Inventory (BFI), and Richard Campbell Sleep Questionnaire (RCSQ) forms were completed. Participants who volunteered for the study were informed that interventions would occur at 8:00 PM if they were in the experimental group. The interventions were implemented for a total of 30 min between 8:00 and 8:30 PM for participants in the experimental group and were carried out consecutively over four days. No interventions were administered to individuals in the control group. To collect data, scales (RCSQ and BFI) were applied to all three groups for five days, half an hour before the interventions (Fig. 2). Information was collected by marking on paper by the researcher through one-on-one face-to-face interviews.

### Implementation of ınterventions

During the study's implementation, care was taken to ensure that there were no external interventions, that the patient's room was calm, and that the individual was not asleep or during mealtime. Since interventions needed to be administered two hours before the patients' general sleep times, it was decided that they would take place at 8:00 PM.

#### Application of foot massage (FM)[14–16]

The foot massage was performed by a nurse holding a massage application certification and a PhD degree. The materials used for the foot massage included paper towels and baby oil. The massage began with odorless baby oil to facilitate the practitioner’s hand manipulation. This was followed by 2.5 min of warming and relaxation movements, after which massage techniques, including friction and petrissage methods, were applied to the toes, soles, tops, lateral surfaces, and medial surfaces of the foot for 10 min. The procedure concluded with another 2.5 min of warming and relaxation movements. Each session lasted a total of 30 min, with 15 min dedicated to the right foot and 15 min to the left foot [see Appendix 1]. The foot massage was applied consecutively over four days.

#### Application of bed bath (BB)[17–22]

To provide the same conditions for all patients and ensure their safety, all patients received a bed bath instead of a standing shower. The bed bath was administered by nurses holding PhD degrees. The materials used for the bed bath included disposable gloves, waterproof protective covers, a face towel, two bath towels, disposable cotton washcloths, water at a temperature of 43–45 °C, a water thermometer (Lirazon Digital Kitchen Thermometer for water: Heat measurement range −50 °C to 300 °C (−58 °F to 572 °F)), a clean set of bed linens, clean underwear, and pajamas. During the bed bath, disposable cotton washcloths were soaked in water at a temperature of 43–45 °C and used to wipe the entire body, followed by drying. The wiping process was carried out in the following order: eyes, forehead, nostrils, cheeks, chin, neck, ears, arms, hands, chest, abdomen, nape, shoulders, back, hips, legs, feet, and perineal area. Each application lasted 30–40 min [see Appendix 1]. The bed bath was also applied consecutively over four days.

### Data analysis

The results of the analyses in this study were interpreted at a significance level of 0.05 and within a 95% confidence interval. The data were analyzed using the SPSS 20 statistical software package. Descriptive statistics, including frequency, percentage, mean, median, and minimum–maximum values, were calculated. Chi-squared tests were employed to compare descriptive characteristics. Mean and standard deviation values were calculated for the participants' scale scores. The normality of scale scores was assessed using the Shapiro–Wilk test and skewness-kurtosis coefficients; it was found that the skewness and kurtosis coefficients fell within the range of + 1.5 [32], and the Shapiro–Wilk test indicated a significance level above 0.05, suggesting that the scores were normally distributed. For the comparison of scale scores among groups, One-Way ANOVA was utilized, while Repeated Measures ANOVA was employed for comparing repeated measurements, and Bonferroni tests were conducted to identify significant differences. Multiple ANCOVA was performed to test whether between-group differences in fatigue and sleep quality were independent of potential confounding factors.

## Results

The average ages of participants were 65.83 ± 7.03 in the Foot Massage (FM) group, 65.5 ± 15.02 in the Bed Bath (BB) group, and 63.53 ± 12.42 in the Control (C) group. No statistically significant difference was found between the groups in terms of age (*p* > 0.05). Gender distribution showed that 50% of the FM group, 58.3% of the BB group, and 60% of the control group were male. Marital status revealed that 83.3% of the FM group, 75% of the BB group, and 80% of the control group were married. Regarding parenthood, all of the participants in the FM group, 91.6% in the BB group, and 93.3% in the control group had children. In terms of educational background, primary school graduates comprised 58.3% of the FM group, 41.7% of the BB group, and 66.6% of the control group. The employment status showed that 41.7% of the FM and BB groups were laborers, compared to 53.3% in the control group. Additionally, 58.3% of participants in the FM and BB groups were retired, while the rate was 46.7% in the control group. No significant differences were identified among the groups regarding sociodemographic characteristics (*p* > 0.05), indicating homogenous distribution (Table 1).

**Table 1:** Sociodemographic characteristics of the participants (*N* = 39)

Characteristics		Total	FM (*n* = 12)	BB (*n* = 12)	C (*n* = 15)	*p* Test value
		Mean ± SD	Mean ± SD	Mean ± SD	Mean ± SD	
Age	(year)	64.91 ± 11.68	65.83 ± 7.03	65.50 ± 15.02	63.53 ± 12.42	.874 F =.135^a^
Height	(cm)	164.97 ± 9.83	166.16 ± 8.05	161.50 ± 9.94	167.07 ± 11.03	.331 F = 1.143^a^
Weight	(kg)	67.70 ± 11.75	72.00 ± 13.86	66.25 ± 9.57	65.07 ± 11.25	.304 F = 1.233^a^
		*n*(%)	*n* (%)	*n* (%)	*n* (%)	*p*
Gender	Female	17 (43.6)	6 (50.0)	5 (41,7)	6 (40.0)	.837 X =.356^b^
	Male	22 (56.4)	6 (50.0)	7 (58.3)	9 (60.0)	
Marital status	Married	31 (79.5)	10 (83.3)	9 (75.0)	12 (80.0)	0.805 X =.435^b^
	Single	8 (20.5)	2 (16.7)	3 (25.0)	3 (20.0)	
Number of children	None	2 (5.1)	-	1 (8.3)	1 (6.7)	.576 X = 4.749^b^
	1	3 (7.7)	2 (16.7)	-	1 (6.7)	
	2	17 (43.6)	5 (41.7)	7 (58.3)	5 (33.3)	
	3 and more	17 (43.6)	5 (41.7)	4 (33.3)	8 (53.3)	
With whom they live	Alone	1 (2.6)	-	-	1 (6.7)	.533 X = 5.081^b^
	Nuclear family	35 (89.7)	10 (83.3)	11 (91.7)	14 (93.3)	
	Relatives	2 (5.1)	1 (8.3)	1 (8.3)	-	
	Nursing home	1 (2.6)	1 (8.3)	-	-	
Education level	Literate	3 (7.7)	2 (16.7)	-	1 (6.7)	.461 X = 5.669^b^
	Primary School	22 (56.4)	7 (58.3)	5 (41.7)	10 (66.6)	
	High School	10 (25.6)	2 (16.7)	5 (41.7)	3 (20.0)	
	University	4 (10.3)	1 (8.3)	2 (16.7)	1 (6.7)	
Job	None	12 (30.8)	4 (33.3)	3 (25.0)	5 (33.3)	.838 X = 4.204^b^
	Farmer	2 (5.1)	1 (8.3)	1 (8.3)	-	
	Labourer	18 (46.2)	5 (41.7)	5 (41.7)	8 (53.3)	
	Officer	5 (12.8)	1 (8.3)	3 (25.0)	1 (6.7)	
	Self-employment	2 (5.1)	1 (8.3)	-	1 (6.7)	
Working status	Working	5 (12.8)	1 (8.3)	1 (8.3)	3 (20.0)	.808 X = 1.605^b^
	Not working	13 (33.3)	4 (33.3)	4 (33.3)	5 (33.3)	
	Retired	21 (53.9)	7 (58.3)	7 (58.3)	7 (46.7)	
	**Total**	39 (100)	12 (100)	12 (100)	15 (100)	

*FM* Foot Massage, *BB* Bed Bath, *C* Control, *SD* Standart Deviation

^a^ One-Way Anova Test, ^b^ Pearson Chi-Square Test

^*^p <.05

Regarding medical characteristics, the average duration since cancer diagnosis was 17.08 ± 32.98 months in the FM group, 6.33 ± 6.3 months in the BB group, and 4.23 ± 4.76 months in the control group. Chemotherapy status showed that 91.7% of the FM group, 100% of the BB group, and 93.3% of the control group were receiving treatment. At the time of the study, 66.7% of the FM group, 83.3% of the BB group, and 80.0% of the control group were actively undergoing chemotherapy. No statistically significant differences were found among the groups concerning medical history (*p* > 0.05) (Table 2).

**Table 2:** Medical status of the participants (*N* = 39)

Status		Total	FM (*n* = 12)	BB (*n* = 12)	C (*n* = 15)	*p* Test value
		Mean ± SD	Mean ± SD	Mean ± SD	Mean ± SD	
Cancer duration (months)		9.08 ± 19.60	17.08 ± 32.98	6.33 ± 6.30	4.23 ± 4.76	.224 F = 1.562^a^
		*n* (%)	*n* (%)	*n* (%)	*n* (%)	*p*
Medical diagnosis	Lung cancer	8 (20.5)	4 (33.3)	1 (8.3)	3 (20)	.211 *X* = 27.019^b^
	Breast cancer	6 (13.4)	1 (8.3)	2 (16.7)	3 (20)	
	Colon cancer	2 (5.1)	2 (16.7)	0	0	
	Stomach cancer	9 (23.1)	2 (16.7)	1 (8.3)	6 (40.0)	
	Pancreas cancer	4 (10.3)	2 (16.7)	2 (16.7)	0	
	Bladder cancer	3 (7.8)	1 (8.3)	1 (8.3)	1 (6.7)	
	Larenks cancer	1 (2.6)	0	1 (8.3)	0	
	Skin cancer	1 (2.6)	0	1 (8.3)	0	
	Over cancer	2 (5.1)	0	2 (16.7)	0	
	Mouth cancer	1 (2.6)	0	1 (8.3)	0	
	Thyroid cancer	1 (2.6)	0	0	1 (6.7)	
	Liver cancer	1 (2.6)	0	0	1 (6.7)	
Treatments received	Chemotherapy	37 (94.9)	11 (91.7)	12 (100)	14 (93.3)	.124 X = 27.245^b^
	Radiotherapy	20 (51.3)	10 (83.3)	9 (75.0)	1 (6.7)	
	Surgery	13 (33.3)	3 (25)	2 (16.7)	8 (46.6)	
	Smart medicine	3 (7.7)	3 (25)	-	-	
The current treatment	Chemotherapy	30 (76.9)	8 (66.7)	10 (83.3)	12 (80)	.328 X = 6.922^b^
	Radiotherapy	2 (5.1)	2 (16.7)	-	-	
	Chemotherapy + Radiotherapy	1 (2.6)	-	1 (8.3)	-	
	General follow-up	6 (15.4)	2 (16.7)	1 (8.3)	3 (20)	

*FM* Foot Massage, *BB* Bed Bath, *C* Control

^a^ One-Way Anova Test, ^b^ Pearson Chi-Square Test

^*^p <.05

Fatigue levels, measured by the Brief Fatigue Inventory (BFI) over five days, showed no significant differences on Day 1 (FM: 6.36 ± 3.06, BB: 8.22 ± 1.52, C: 6.16 ± 2.61; *p* > 0.05). However, starting from Day 2, the FM group showed significantly lower fatigue scores (4.47 ± 3.1) compared to the BB group (7.19 ± 1.71) (*p* < 0.05). On Day 3, the FM group (3.82 ± 2.63) had significantly lower scores than both the BB group (6.56 ± 1.73) and the control group (6.97 ± 2.2) (*p* < 0.05). Similar trends continued on Day 4 (FM: 3.25 ± 2.36, BB: 5.75 ± 2.22, C: 7.58 ± 2.07) and Day 5 (FM: 3.07 ± 2.23, BB: 5.38 ± 2.12, C: 7.87 ± 2.11), with the FM group consistently reporting significantly less fatigue (*p* < 0.001). The FM group also showed significantly lower scores than the BB group on Day 5 (*p* < 0.001). Within-group comparisons revealed a decreasing trend in fatigue scores over time for the FM and BB groups, while the control group showed an increasing trend (*p* < 0.001) (Table 3).

**Table 3:** Comparison of participants' mean BFI scores (*N* = 39)

	Total	FM	BB	C	*p* Test value *Bonferroni*
	Mean ± SD	Mean ± SD	Mean ± SD	Mean ± SD	
Day 1	6.89 ± 2.59	6.36 ± 3.06	8.22 ± 1.52	6.16 ± 2.61	.092 F = 2.563^a^
Day 2	6.08 ± 2.59	4.47 ± 3.10	7.19 ± 1.71	6.55 ± 2.14	.022^*^ **F = 4.270**^**a**^ ***(FM* < *BB)*** ***(FM* < *C)***
Day 3	5.81 ± 2.58	3.82 ± 2.63	6.56 ± 1.73	6.97 ± 2.20	.002^*^ **F = 7.244**^**a**^ ***(FM* < *BB)*** ***(FM* < *C)***
Day 4	5.58 ± 2.81	3.25 ± 2.36	5.75 ± 2.22	7.58 ± 2.07	*p* <.001^**^ **F = 11.926**^**a**^ ***(FM* < *BB)*** ***(FM* < *C)***
Day 5	5.51 ± 2.89	3.07 ± 2.23	5.38 ± 2.12	7.87 ± 2.11	*p* <.001^**^ **F = 15.433**^**a**^ ***(FM* < *BB* < *C)***
	***p*** **Test value**	*p* <.001^**^ **F = 39.876**^**b**^	*p* <.001^**^ **F = 175.263**^**b**^	*p* <.001^**^ **F = 134.691**^**b**^	
	***Bonferroni***	***5* < *1,3***	***4,5* < *1,2*** ***5* < *3***	***1,2* < *4,5*** ***3* < *5***	

*FM* Foot Massage, *BB* Bed Bath, *C* Control

^a^ One-Way Anova Test, ^*b*^*Repeated Measurement Anova Test*

^*^p < .05, ^**^p < *.001*

Sleep quality, assessed with the Richard Campbell Sleep Questionnaire (RCSQ), revealed no significant differences among the groups on Day 1 (FM: 39 ± 25.52, BB: 39.20 ± 14.17, C: 53.53 ± 13.39; *p* > 0.05). On Day 2, scores improved in both intervention groups (FM: 63.83 ± 21.00, BB: 60.58 ± 28.11). From Day 3 onwards, the FM group consistently showed significantly higher sleep quality scores compared to the control group (*p* < 0.05 and *p* < 0.001). Beginning on Day 4, the BB group also showed significantly better scores than the control group (*p* < 0.001). No statistically significant differences were found between the FM and BB groups in RCSQ scores (*p* > 0.05). Within-group comparisons showed an upward trend in sleep quality in both FM and BB groups, while the control group exhibited a decline over the five days (*p* < 0.001) (Table 4).

**Table 4:** Comparison of Participants' Mean RCSQ Scores (*n* = 39)

	FM	BB	C	p Test value *Bonferroni*
	Mean ± SD	Mean ± SD	Mean ± SD	
Day 1	39.00 ± 25.52	39.20 ± 14.17	53.53 ± 13.39	.900 F = 2.593^a^
Day 2	63.83 ± 21.00	60.58 ± 28.11	55.69 ± 20.89	.630 F = 0.468^a^
Day 3	69.91 ± 15.92	56.00 ± 23.51	43.10 ± 17.53	.006^*^ **F = 6.069**^a^ ***FM* > *C***
Day 4	77.50 ± 17.81	65.16 ± 19.44	43.58 ± 17.22	*p* <.001^**^ **F = 11.263**^a^ ***FM* > *C*** ***BB* > *C***
Day 5	82.98 ± 13.37	68.25 ± 18.49	39.53 ± 16.12	*p* <.001^**^ **F = 23.578**^a^ ***FM* > *C*** ***BB* > *C***
p Test value *Bonferroni*	*p* <.001^**^ **F = 239.711**^b^ ***1* < *3,4,5*** ***2* < *4,5***	*p* <.001^**^ **F = 134.547**^b^ ***1,3* < *4,5***	*p* <.001^**^ **F = 147.145**^b^ ***5* < *1*** ***3,4,5* < *2***	

*FM* Foot Massage, *BB* Bed Bath, *C* Control,^a^ One-Way Anova Test, ^*b*^Repeated Measurement Anova Test,^*^p < .05, ^**^p < *.001*

Multiple ANCOVA was performed to test whether between-group differences in fatigue and sleep quality were independent of potential confounding factors, including age, gender, medical diagnosis, cancer duration, and current treatment. The analysis revealed significant group differences in both fatigue levels (F(2,27) = 12.341, *p* < 0.001, ηp2 = 0.478) (Table 5) and sleep quality (F(2,27) = 6.892, *p* = 0.004, ηp2 = 0.338) (Table 6).

**Table 5:** Fatigue Scores by Group After Controlling for Covariates

	FM (*n* = 12)	BB (*n* = 12)	Control (*n* = 15)	F	*p*	ηp^2^
Adjusted mean (SE)	4.42 (0.48)	7.13 (0.48)	6.84 (0.46)	12.341	** <.001**	.478
95% CI	[3.43, 5.41]	[6.14, 8.12]	[5.89, 7.79]			
*Post-hoc comparisons*						
vs. FM	-	***p* =.001**	***p* =.002**			
vs. BB	***p*** **=.001**	-	***p*** = 1.000			
vs. Control	4.42 (0.48)	7.13 (0.48)	6.84 (0.46)	12.341	** <.001**	.478

Multiple ANCOVA with covariates: age, gender, medical diagnosis, cancer duration, current treatment. Bonferroni-adjusted p-values

**Table 6:** Sleep Qality by Group After Controlling for Covariates

	FM (*n* = 12)	BB (*n* = 12)	Control (*n* = 15)	F	*p*	ηp^2^
Adjusted mean (SE)	66.9 (4.21)	60.2 (4.21)	46.5 (4.05)	6.892	**.004**	.338
95% CI	[58.3, 75.5]	[51.6, 68.8]	[38.3, 54.7]			
*Post-hoc comparisons*						
vs. FM	-	***p*** =.248	***p* =.002**			
vs. BB	*p* =.248	-	***p* =.035**			
vs. Control	***p* =.002**	***p* =.035**	-			

Note*:* Multiple ANCOVA with covariates: age, gender, medical diagnosis, cancer duration, current treatment. Bonferroni-adjusted p-values

Post-hoc comparisons for fatigue indicated that Group FM reported significantly lower fatigue scores than both Group BB (*p* = 0.001, d = 1.64) and the Control group (*p* = 0.002, d = 1.46). No significant difference was found between Group BB and the Control group (*p* = 1.000) (Table 5). Regarding sleep quality, both Group FM and Group BB reported significantly higher scores compared to the control group (Table 6).

## Discussion

Fatigue in cancer patients is a painful, persistent, subjective, physical, emotional or cognitive problem that is not related to recent activity and impairs daily functioning [33]. Sleep problems in cancer patients are not attributable to a single cause, but rather to a combination of issues such as disruption of the body's biological rhythm, chemical changes caused by treatments, and the psychological burden of the disease [33–35]. This study investigated the effects of foot massage (FM) and bed bathing (BB) on fatigue and sleep quality among hospitalized cancer patients, finding partial support for the study hypotheses. For the first four hypotheses (H1^1^–H1^4^), the results indicated that both FM and BB significantly reduced fatigue and improved sleep quality in cancer patients, confirming these hypotheses. Specifically, fatigue levels in the FM group were significantly lower than those in the BB and control groups from the second day onward, while the BB group showed significantly reduced fatigue by the fifth day compared to the control group. These findings support H1^1^ and H1^3^, suggesting both interventions are effective, with FM offering more rapid relief from fatigue. Regarding sleep quality, participants in the FM group demonstrated significantly better sleep quality than those in the control group starting from the third day. Similarly, the BB group showed improved sleep quality by the fourth day. These results affirm H1^2^ and H1^4^, highlighting the positive impact of both FM and BB on sleep quality among cancer patients. Conversely, the fifth and sixth hypotheses (H1^5^ and H1^6^), which posited significant differences in fatigue and sleep quality between the FM and BB groups, were not supported. While FM appeared to provide faster improvements, the overall differences between the two techniques were not statistically significant at the end of the intervention period. This suggests that, although both methods are effective, neither is clearly superior in their overall impact on fatigue and sleep quality. These findings imply that FM and BB, while differing in delivery and mechanism, offer similar therapeutic benefits in supportive cancer care. Therefore, the choice between them can be guided by patient preference, feasibility, and available resources. The results of our research align with findings reported in studies by Akyuz Ozdemir and Can (2021) [4], Hesami et al. (2019)[36], Mazloum et al. (2023)^17^, and Ünal Aslan (2022) [37]. For instance, Hesami and colleagues found that foot reflexology massage significantly reduced fatigue, while Mazloum's study indicated that both foot massage and warm foot baths effectively alleviated fatigue among radiotherapy patients. Our current study, examining the effects of FM and BB on reducing fatigue in cancer patients, is consistent with existing literature and supports the clinical significance of FM in alleviating fatigue. Consistent with the literature, our study found that BB has a limited effect on fatigue. Additionally, improvements in sleep quality were noted for both the FM and BB groups. Our study also aligns with findings by Haghayegh et al. (2019)[38], which indicated the positive effects of bathing practices on sleep. As noted in the systematic review by Nasiri et al. (2024)[39], foot baths above 40 °C are effective in enhancing sleep quality. A study examining the effects of warm salt water and warm water baths applied to the hands and foot of patients with rheumatoid arthritis on fatigue and sleep quality indicated that both baths were effective [40]. While the Mindfulness-Based Cancer Recovery programme was found to be a meaningful and effective method for reducing cancer-related fatigue, it was observed to have no effect on sleep quality [35]. It has been noted that this situation may stem from the individual differences among participants. According to a meta-analysis study, comprehensive sleep interventions are more effective than single sleep interventions [33]. Although both interventions are non-pharmacological and cost-effective with minimal risk of adverse effects, they are not necessarily "simple" procedures. Their proper application requires appropriate training and standardization to ensure patient safety and maximize benefit. Future studies should continue exploring these interventions over extended periods and across different cancer types and treatment phases to determine their long-term effectiveness and applicability in broader settings.

Our study demonstrated that both FM and BB interventions led to a significant reduction in fatigue and improvement in sleep quality in cancer patients. These findings align with the proposed physiological mechanisms of sleep-focused interventions, which are thought to promote relaxation, activate the parasympathetic nervous system, and support overall psychophysical balance, thereby mitigating symptoms like fatigue and insomnia [33]. The beneficial effects observed in our sample are particularly noteworthy given the complex clinical context of cancer. Fatigue and sleep disturbances are highly prevalent in this population and are strongly influenced by factors such as cancer type, treatment modalities (e.g., chemotherapy, radiotherapy), and time since diagnosis [41, 42]. For instance, patients with colorectal cancer may report greater symptom burden,[43] and insomnia is frequently cited as a side effect of chemotherapy [34]. Fatigue is a common side effect of cancer treatments like chemotherapy, radiation therapy, and surgery [33, 35]. In a descriptive study, it was noted that fatigue levels or sleep quality did not change according to the type of treatment [43]. Some studies report that female cancer patients have a higher rate of poor sleep quality compared to males [34, 35]. However, a specific study conducted in Iran found that fatigue levels were significantly higher in male patients than in female patients [43]. In our study, it was observed that the type of treatment, age, gender, medical diagnosis or cancer duration did not alter the effect of interventions on sleep quality or fatigue. Furthermore, it is possible that our study was not sufficiently powered to detect small subgroup effects.

### Limitations

This study has several limitations that should be considered when interpreting the findings. First, sleep hygiene practices are inherently individual and vary widely. Since these routines were not standardized or controlled across participants, individual variations in sleep-related behaviors may have influenced the outcomes. Second, although the interventions were scheduled before bedtime, other factors during hospitalization may have affected participants’ fatigue and sleep levels. For example, daily clinical procedures, such as imaging (e.g., radiography, magnetic resonance imaging), were not standardized across participants, potentially causing variability in daily physical and mental fatigue. Third, although the calculated sample size has been reached, the number of participants is still small. Fourth, there was no blinding at any stage of the study. However, bias was attempted to be controlled using standardized, self-reported outcome measures.

## Conclusion

This study indicates that foot massage and bed baths may have beneficial short-term effects in reducing fatigue and improving sleep quality among hospitalized cancer patients. The study suggests the potential value of integrating basic comfort-based interventions into routine nursing care. In this context, structured in-service training programs may support nurses in delivering these interventions safely and consistently as part of supportive cancer care. Future research is recommended to examine the long-term effects of these interventions in more controlled settings and diverse patient populations, including those receiving care at home.

## Supplementary Information

Below is the link to the electronic supplementary material.

Supplementary file1 (DOCX 18 KB)

## Data Availability

No datasets were generated or analysed during the current study.
