# Supplementary material for: The effectiveness of nursing interventions on fatigue and sleep quality in hospitalized cancer patients: the role of foot massage and bed bath
Source: Support Care Cancer. 2026 Feb 5;34(3):169. doi: 10.1007/s00520-026-10386-7 (PMC12876477; doi:10.1007/s00520-026-10386-7)
Supplement: Supplementary file 1 — Supplementary file1 (DOCX 18 KB) [file 520_2026_10386_MOESM1_ESM.docx]

***Application of Foot Massage:***

| **No** | **Application Steps of Foot Massage** |
| --- | --- |
|  | The individual was informed about the procedure, |
|  | Hands washed, |
|  | In order for the individual to be in a comfortable position, it was ensured that he/she was in a lying or semi-sitting position, taking into account his/her limitations, |
|  | We crossed the toe of the individual and sat so that the individual's feet were at our chest level when we sat down, |
|  | The hands were warmed by rubbing them together so that they were not cold, |
|  | According to the size of the foot, an amount of non-allergenic baby oil was taken to slightly moisturise the foot, and spread on the foot to provide lubrication, |
|  | 2.5 minutes of foot warming and relaxation movements were performed,   - After the heel of the right foot was fixed with the left hand, the region where the patient's big toe was located was grasped with the thumb of the right hand, and after the foot was fixed in this way, the ankle was rotated first to the right and then to the left with the right hand, - The Achilles tendon was grasped with the left hand, the toe was held with the right hand and moved up and down three times, - The top of the foot was grasped with both hands and rotated left and right, - After this, the entire foot (including all toes and knuckles) was rotated first to the right and then to the left. |
|  | All parts of the foot were massaged with friction and petrissage method for ten minutes;2 dakika, ayak parmaklarına   - 2 minutes, toes - 2 minutes, to the lower sole of the foot - 2 minutes, to the upper surface of the foot - 2 minutes, to the outer edges of the foot - 2 minutes, to the inner edges of the foot |
|  | 2.5 minutes of foot heating and relaxation movements were performed |
|  | In order not to lose the warmth of the individual's foot, it was covered or socks were put on, and the left foot was switched to the left foot without a break. |
|  | The same applications were made on the left foot. |
|  | When the massage was over, the individual was helped to take a comfortable position. |
|  | Hands were washed. |
|  | The individual was given a comfortable position. The call button and special tools were placed next to the individual. |

***Application of the Bed Bath:***

| **No** | **Application Steps of Bed Bath** |
| --- | --- |
|  | Materials: Disposable gloves, waterproof protective covers, a face towel, two bath towels, disposable cotton washcloths, water at a temperature of 43-45 °C, a water thermometer (Lirazon Digital Kitchen Thermometer for water: Heat measurement range -50 °C up to 300 °C (-58 °F up to 572 °F)), a clean set of bed linens, clean underwear, and pajamas. |
|  | Explained the process, obtained the individual's authorisation |
|  | The window/door was closed to ensure privacy and safety and to prevent the individual from getting cold. |
|  | The need for elimination was questioned, and if necessary, the need for elimination was provided first. |
|  | Hands were washed, gloves were worn. |
|  | The clothes of the individual were removed from under the bedsheet. |
|  | The pillow was taken and the head end of the bed was raised 30-45 degrees. |
|  | Disposable cotton wipes were wetted with 43-45 °C water. |
|  | Eye and face cleaning was performed.  *Eye cleaning (starting from the eye on the far side first) from the inner canthus to the outer canthus,*  *Face cleaned (from the centre to the outer edges: Forehead, Nose sides, Cheeks, Chin, Neck and Ears) were wiped wet and dried with a towel.* |
|  |  |
|  | Bath towel was laid lengthwise under the arm |
|  | The arms were applied with a wet cotton wipe from the wrist upwards, from far to near, and dried with a towel. |
|  | Hands were applied with a wet cotton wipe and dried with a towel |
|  | The chest area were applied with a wet cotton wipe and dried with a towel. The abdominal area were applied with a wet cotton wipe and dried with a towel |
|  | The individual was given a side or prone position |
|  | Nape, shoulders, back and buttocks were applied with a wet cotton wipe and dried with a towel |
|  | Legs were applied with a wet cotton wipe in the direction of venous circulation from far to near, and dried with a towel. |
|  | Feet were applied with a wet cotton wipe and dried with a towel |
|  | The individual was turned on his/her back, the genital area were applied with a wet cotton wipe and dried with a paper napkin. |
|  | At the end of the procedure, bed linen and bedding were changed if necessary. |
|  | Hands were washed, the materials used were trashed. |
|  | The individual was given a comfortable position. The call button and special tools were placed next to the individual. |
